# Epitope-specific immunity against *Staphylococcus aureus* coproporphyrinogen III oxidase

**DOI:** 10.1038/s41541-020-00268-2

**Published:** 2021-01-18

**Authors:** Alexander Klimka, Sonja Mertins, Anne Kristin Nicolai, Liza Marie Rummler, Paul G. Higgins, Saskia Diana Günther, Bettina Tosetti, Oleg Krut, Martin Krönke

**Affiliations:** 1 Institute for Medical Microbiology, Immunology and Hygiene, University Hospital Cologne, Cologne, Germany; 2 German Center for Infection Research (DZIF), Partner site Bonn-Cologne, Cologne, Germany; 3 Cologne Cluster of Excellence on Cellular Stress Responses in Aging-Associated Diseases (CECAD), Cologne, Germany; 4 Center for Molecular Medicine Cologne (CMMC), Cologne, Germany; 5 Present Address: Paul-Ehrlich Institute, Langen, Germany

**Keywords:** Vaccines, Peptide vaccines, Immunology, Microbiology

## Abstract

*Staphylococcus aureus* represents a serious infectious threat to global public health and a vaccine against *S. aureus* represents an unmet medical need. We here characterise two *S. aureus* vaccine candidates, coproporphyrinogen III oxidase (CgoX) and triose phosphate isomerase (TPI), which fulfil essential housekeeping functions in heme synthesis and glycolysis, respectively. Immunisation with rCgoX and rTPI elicited protective immunity against *S. aureus* bacteremia. Two monoclonal antibodies (mAb), CgoX-D3 and TPI-H8, raised against CgoX and TPI, efficiently provided protection against *S. aureus* infection. MAb-CgoX-D3 recognised a linear epitope spanning 12 amino acids (aa), whereas TPI-H8 recognised a larger discontinuous epitope. The CgoX-D3 epitope conjugated to BSA elicited a strong, protective immune response against *S. aureus* infection. The CgoX-D3 epitope is highly conserved in clinical *S. aureus* isolates, indicating its potential wide usability against *S. aureus* infection. These data suggest that immunofocusing through epitope-based immunisation constitutes a strategy for the development of a *S. aureus* vaccine with greater efficacy and better safety profile.

## Introduction

*Staphylococcus aureus* (*S. aureus*) is associated with a significant disease burden causing life-threatening diseases, such as deep wound infections, bacteremia, endocarditis, pneumonia, osteomyelitis, and enterotoxin-mediated shock^1^. Antibiotic resistance, specifically methicillin-resistant *Staphylococcus aureus* (MRSA), is widespread and of aggravating concern. Although vaccination strategies against *S. aureus* have attracted much attention in basic and clinical research, no *S. aureus* vaccine is currently available^2–4^. Specific challenges to development of a *S. aureus* vaccine include low immunogenicity of pathogen-derived antigens, a lack of natural immunity to *S. aureus*, multiple virulence and immune evasion factors as well as redundant nutrition acquisition pathways. All of these challenges compromise a straightforward strategy to delineate a correlate of protection.

In general, neutralising antibodies inhibiting pathogen interaction with or entry into host cells or detoxifying virulence factors represent a dominant principle of protection provided by vaccines. For instance, due to nasal colonization most adult humans have high levels of circulating antibodies against many staphylococcal antigens which seem to provide some protection against invasive infection with *S. aureus*^5,6^. Classical vaccine approaches, targeting *S. aureus* toxins for neutralisation or surface antigens for production of opsonising antibodies, have not worked against *S. aureus* in clinical trials. Similarly, the targeting of *S. aureus* proteins serving important roles in host-pathogen interactions, including adhesion to host cells, binding to and degradation of extracellular matrix proteins, iron-uptake or intervention with the host fibrinolytic system remained unsuccessful^2–4^. Preclinical and clinical data repetitively indicate that although immunisation with *S. aureus* antigens usually results in high antibody titers, this does not confer protection against *S. aureus* infections^7^.

Induction of a high-titered antibody response by a vaccine is not tantamount to protection and may even be detrimental by causing immune enhancement of disease, which is well known for vaccines against viral pathogens. For example, Song and coworkers recently identified a linear B-cell epitope on the prM protein of dengue virus as a major immunodominant B-cell epitope involved in antibody-dependent enhancement of dengue virus infection^8^. Although vaccine-mediated immune enhancement has not been an obvious safety concern for *S. aureus* vaccine development, the knowledge of protective, non-protective and disease enhancing B-cell epitopes represents a strategy for refined vaccine development. In this respect, the use of monoclonal antibodies (mAbs) to design new vaccines has been previously proposed by Burton^9^. Monoclonal Abs are now an integral part of the ‘reverse vaccinology 2.0’ concept^10,11^, where mAbs are used to distinguish protective from non-protective epitopes and to support immunofocused antigen design. An epitope-focused vaccine is anticipated to improve its immunogenic precision level, resulting in a vaccine with a greater efficacy and safety profile. Indeed, an epitope-focused strategy has been successfully employed for the development of a vaccine against RSV that has resisted traditional vaccine development in the past^12^.

We here targeted two non-redundant *S. aureus* housekeeping proteins, coproporphyrinogen III oxidase (CgoX) and triose phosphate isomerase (TPI), which are essential for heme synthesis and glycolysis, respectively. Staphylococcal CgoX (EC: 1.3.3.15, also known as HemY) catalyses the oxidation of coproporphyrinogen III to coproporphyrin III^13,14^ but can also oxidise protoporphyrinogen IX to protoporphyrin IX^15^. Indeed, identical aa sequences are deposited for *S. aureus* CgoX and protoporphyrinogen oxidase in public data banks like UniprotKB (Uniprot.org, compare Q2FXA5 and A0A0H3K8Y5). The CgoX-mediated generation of protoporphyrin IX, but not coproporphyrin III, is stimulated by heme-bound HemQ, which is mediated by superoxide^14^. TPI catalyses the reversible interconversion of the triose phosphate isomers dihydroxyacetone and D-glyceraldehyde 3-phosphate. It plays an important role in glycolysis and is essential for efficient energy production. CgoX and TPI were previously identified within a group of anchorless cell wall *S. aureus* proteins^16^. The surface location of CgoX and TPI suggests additional functions beyond their role in cellular homeostasis, corresponding to the group of covalently cell wall anchored (CWA) proteins, several of which being multifunctional and involved in *S. aureus* pathogenesis^17^. Indeed, TPI has been suggested to have plasminogen binding activity, which might be relevant to staphylococcal invasion^18,19^. In contrast, whereas the intracellular role of CgoX in heme synthesis is well known, an extracellular function of CgoX has not yet been described. Clearly, the intracellular non-redundant and essential action of CgoX and TPI in *S. aureus* homeostasis is not accessible for antibodies. Thus, vaccinal targeting of their putative extracellular action is not expected to push directly the development of escape mutants. We here show that immunisation with recombinant (r) CgoX or rTPI protects mice from *S. aureus* bacteremia. Correspondingly, mAbs raised against CgoX and TPI significantly improved survival in a murine sepsis model. Furthermore, a short, 12 aa linear epitope specifically recognised by a protective CgoX-D3 mAb is demonstrated to provide highly efficient protection against *S. aureus* infection when used for active immunisation.

## Results

### Selection of CgoX and TPI as vaccine candidates

**Fig. 1:**
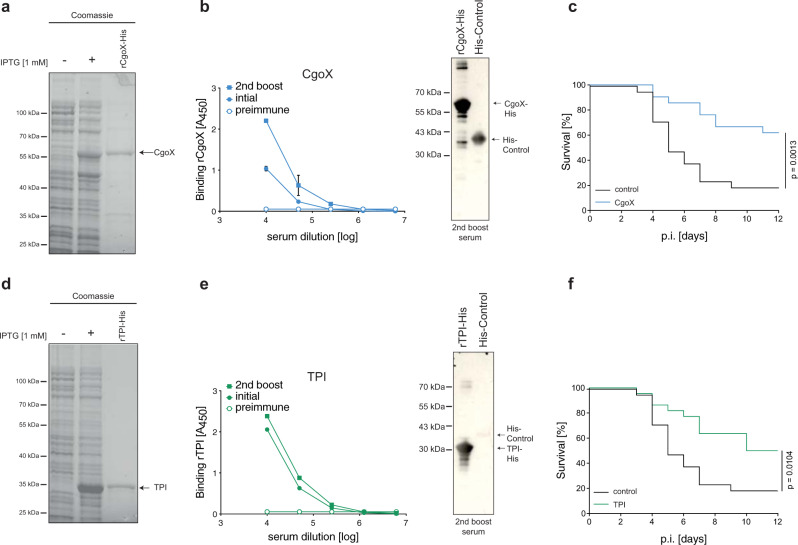
Active immunisation with *S. aureus* vaccine candidates. **a** SDS-PAGE analysis of coomassie stained *E. coli* lysate and purified rHis_6_-tagged CgoX protein (56 kDa) expressed in *E. coli* BL21(DE3). DE3 cells were left untreated (−) or induced with IPTG (+) for expression. **b** Left panel: antigen-specific IgG response of immunised BALB/c mice. Sera of mice (*n* = 12) immunised with *S. aureus* rCgoX formulated with Freund’s adjuvant (FA) were collected and pooled at day 0 (preimmune), 14 (initial) and 66 (2nd boost) after initial immunisation and analysed by ELISA for IgG binding to rCgoX-His (*n* = 2). Data are presented as mean ± s.d. Right panel: antigen-specificity of sera from immunised mice was analysed by WB. Sera collected and pooled at day 66 after initial immunisation with rCgoX-His were analysed for binding to rCgoX-His or His-tagged *S. aureus* rPephyd protein (39 kDa) used as control. Bound murine IgG were detected with anti-mIgG-HRP. **c** Survival of immunised mice after challenge with *S. aureus* (*n* = 11 for control, *n* = 12 for CgoX). BALB/c mice were immunised with rCgoX-His/FA and challenged i.v. with 3 × 10^7^ *S. aureus* strain ATCC 29213. Control mice were immunised with BSA/FA. Significance was calculated by Log-rank (Mantel-Cox) test. **d** SDS-PAGE analysis of coomassie stained *E. coli* lysate and purified His_6_-tagged TPI protein (32 kDa) expressed in *E. coli* BL21(DE3). DE3 cells were left untreated (−) or induced (+) with IPTG for expression. **e** BALB/c mice (*n* = 12) were immunised with *S. aureus* antigen rTPI-His/FA and serum pools were analysed by ELISA for IgG binding to rTPI-His (*n* = 2). Data are presented as mean ± s.d. Right panel: Sera collected and pooled at day 66 after initial immunisation with rTPI-His were analysed by WB for binding to rTPI-His and rPephyd-His (39 kDa) used as control. Bound murine IgG were detected with anti-mIgG-HRP. **f** Survival of mice immunised with rTPI-His formulated with FA (*n* = 11) or BSA / FA (*n* = 12) and challenged i.v. with 3 × 10^7^ *S. aureus* strain ATCC 29213. Significance was calculated by Log-rank (Mantel-Cox) test.

We have previously reported on 37 anchorless cell wall associated *S. aureus* proteins, recognised by naturally occurring antibodies in healthy humans that have potential to serve as new candidates for a protein-based *S. aureus* vaccine. Indeed, some of these targets induced protective immunity against some laboratory *S. aureus* strains when tested in a murine sepsis model^16,20^. Extended testing of this group revealed two further vaccine candidates, coproporphyrinogen III oxidase (CgoX, formerly known as protoporhyrinogen oxidase) and Triose phosphate isomerase (TPI) (Fig. 1). For immunisation studies, recombinant staphylococcal CgoX, and TPI were expressed as His_6_-tagged proteins in *E. coli* and purified by affinity chromatography. The purity and integrity of His_6_-tagged CgoX and TPI were controlled by SDS-PAGE (Fig. 1a, d). Mice were immunised i.p. with 80 µg of recombinant protein and Freund´s adjuvant and boosted with 40 µg antigen in incomplete Freund’s adjuvant s.c. at days 33 and 56. Immunisation with rCgoX and rTPI induced high titers of IgG antibodies recognising the respective recombinant *S. aureus* protein (Fig. 1b, e). Western blot analysis revealed that the antibody response against CgoX was partially directed against the His_6_-tag (Fig. 1b). Eight days after the second boost, mice were challenged i.v. with with the methicillin-sensitive *Staphylococcus aureus* (MSSA) strain ATCC29213 (day 64). Immunisation with rCgoX and rTPI induced significant protection against *S. aureus* infection in a murine sepsis model (Fig. 1c, f). CgoX and TPI have orthologs in mouse and man. Staphylococcal CgoX shared 23% identity with its human and mouse ortholog, PPOX. Staphylococcal TPI showed 22% identities with its ortholog in mouse and man (Supplementary Fig. 1a, b). Thus, when used as whole antigen for immunisation, these proteins bear a remote risk of eliciting antibodies cross-reacting with human orthologs.

### Generation of protective antibodies

**Fig. 2:**
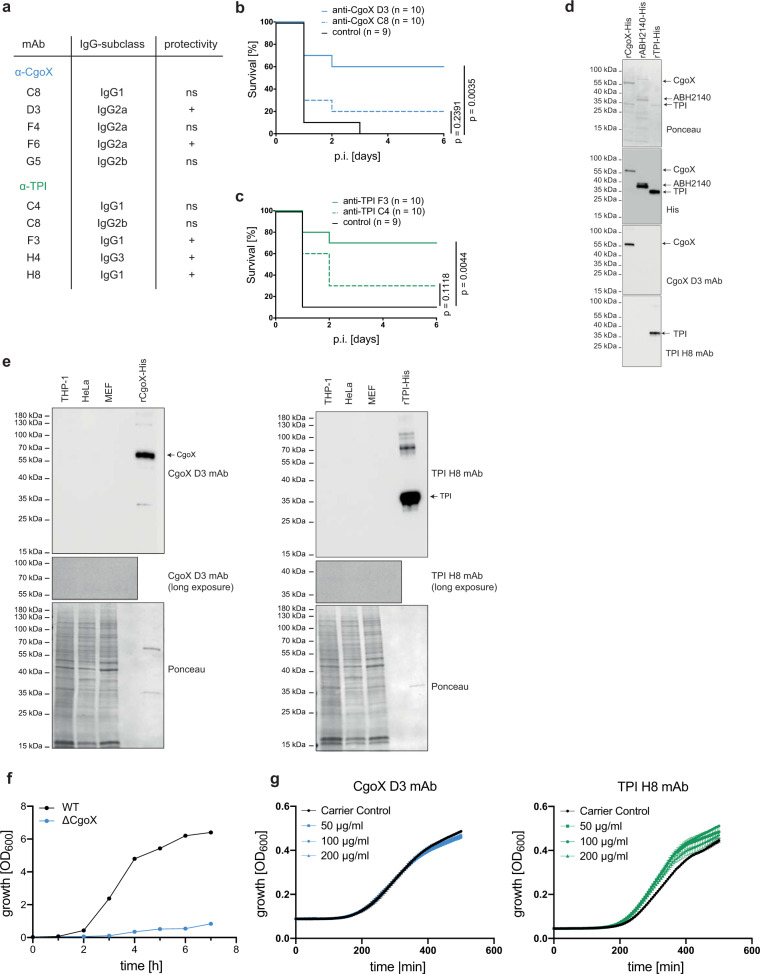
Passive immunisation with mAbs against *S. aureus*. **a** Panel of mAbs obtained after immunisation of BALB/c mice with *S. aureus* rCgoX and rTPI. Protectivity was evaluated after passive immunisation and subsequent challenge of BALB/c mice (*n* = 10) with a lethal dose of *S. aureus* ATCC 29213. **b** BALB/c mice were passively immunised i.p. with 200 µg of the indicated mAbs (or with PBS as control) and subsequently challenged with 1 × 10^6^ cfu *S. aureus* strain USA300 i.p. Significance was calculated by Log-rank (Mantel-Cox) test. **c** BALB/c mice were passively immunised i.p. with 300 µg of the indicated mAbs (or with PBS as control) and subsequently challenged with 5 × 10^5^ cfu *S. aureus* strain ATCC 29213 i.p. Significance was calculated by Log-rank (Mantel-Cox) test. **d** Specific binding of mAbs to their corresponding recombinant antigen. A His_6_-tagged unrelated *S. aureus* protein, rABH2140-His, was used as control). **e** Cross reactivity analysis of mAbs with human and murine cell lysates. 20 ng of purified, rCgoX or rTPI (lane 1), 2 µg of HeLa cell lysate (lane 2), 2 µg of THP-1 cell lysate (lane 3) and 2 µg of MEF cell lysate (lane 4) were separated by SDS-PAGE and analysed by staining with indicated mAbs. **f** Representative proliferation study of WT and CgoX-deficient (ΔC*goX*) *S. aureus* strain USA300 JE2. **g** Growth curves of *S. aureus* strain USA300 JE pre-incubated with the indicated mAbs (*n* = 4 biological replicates). Experiments are respresentatives of three independent experiments.

To dissect protective from non-protective epitopes of *S. aureus* antigens, mAbs were raised against CgoX and TPI by standard hybridoma technology^20^. For each antigen, protective and non- or less-protective mAbs were identified (Fig. 2a). The protective efficacy of mAb CgoX-D3 and mAb TPI-H8 is demonstrated in a murine sepsis model with either the MSSA strain ATCC29213 or MRSA USA300 (Fig. 2b, c). Of note, all mAbs showed protection at doses between 200 µg and 300 µg per mouse (Supplementary Fig. 2c) and worked equally well for MSSA and MRSA strains (Fig. 2b, c and Supplementary Fig. 2a, b). MAb CgoX-D3 and mAb TPI-H8 specifically recognised the respective recombinant protein (Fig. 2d). The heavy chain subtypes isolated for the two *S. aureus* antigens were predominantly IgG1. The protective mAbs CgoX-D3 and TPI-H8 did not cross-react with their respective human orthologs (Fig. 2e). CgoX and TPI are essential intracellular housekeeping enzymes involved in heme synthesis and glycolysis, respectively. Indeed, the genetic ablation of *cgoX* attenuated *S. aureus* proliferation (Fig. 2f), whereas *tpiA* deletion *S. aureus* mutants could not be generated suggesting that TPI is essential for *S. aureus* growth. Given that antibodies do not pass the *S. aureus* plasma membrane, the protective effects of monoclonal CgoX-D3 and TPI-H8 antibodies should be unrelated to intracellular functions of their respective target antigen. Indeed, none of the mAbs inhibited *S. aureus* proliferation in vitro (Fig. 2g).

It is noteworthy that humanisation of the murine mAbs CgoX-D3 and TPI-H8 preserved their antigen specificity and function, i.e. recognition of their target antigen and in vivo protectiveness against *S. aureus* USA300 (Supplementary Fig. 2a–d), suggesting that their mode of action is not dependent on interactions with the murine Fc fragment or distinct IgG subclasses. Like the murine mAbs, humanised mAbs (huMAbs) CgoX-D3 and TPI-H8 did not inhibit in vitro proliferation of *S. aureus* (Supplementary Fig. 2e, f). Moreover, CgoX-D3 and TPI-H8 provide only marginal opsonizing activity of *S. aureus* by human neutrophils or murine macrophages, respectively (data not shown).

### Epitope mapping of protective and non-protective monoclonal antibodies

**Fig. 3:**
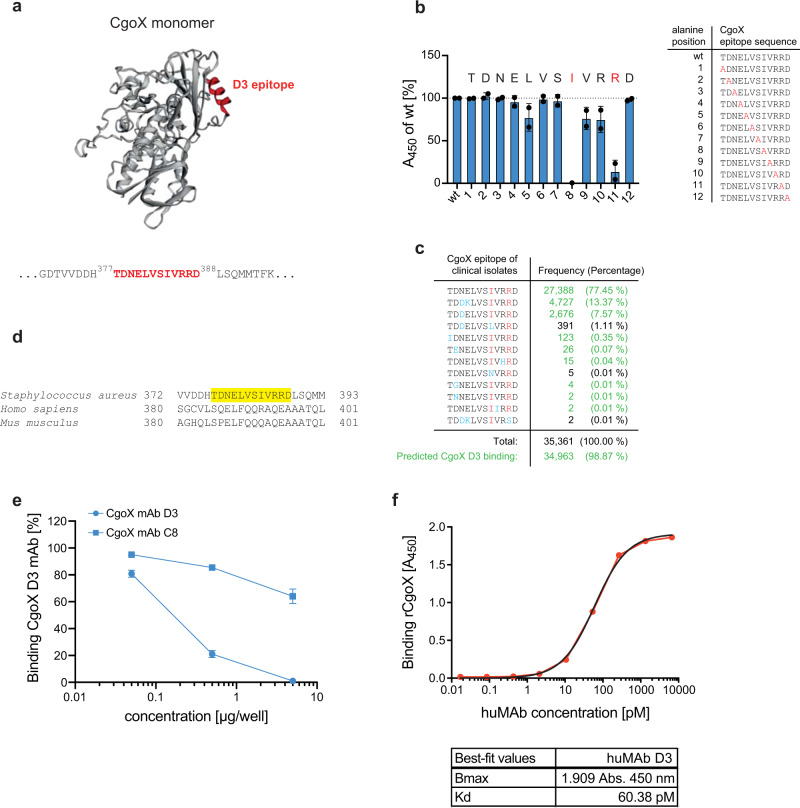
Characterisation of anti-CgoX mAb D3 epitope. **a** *S. aureus* CgoX structure was modulated from *B. subtillis* (3I6D.pdb). The linear epitope of mAb D3 (red) was identified by microarray technology using overlapping 13mer CgoX peptides (Supplementary Fig. 3). Protein structure was visualised by EzMol2.1. **b** Alanine scan of epitope peptides for binding analysis of anti-CgoX mAb D3. Single amino acid positions of the D3 epitope were consecutively replaced by alanine (red in right panel). Immobilised peptides were stained by anti-CgoX mAb D3 detected with anti-mIgG-HRP. Data are presented as mean ± s.d. (*n* = 2 technical replicates). **c** Allele frequencies of anti-CgoX mAb D3 epitope. Genome sequences of *S. aureus* clinical isolates were analysed for epitope aa sequence using the RidomSeqsphere core genome multi locus sequence typing (cgMLST) database. Amino acids interacting with paratope of anti-CgoX mAb D3 according to alanine scan are marked in red. Amino acid differences from identified epitope peptide sequence are marked in blue. Frequencies of alleles with non-restricted binding of anti-CgoX mAb D3 are marked in green. **d** Uniqueness of the CgoX D3 epitope in *S. aureus*. Sequence alignment of CgoX from *S. aureus* with PPOX from *H. sapiens* and *M. musculus*. CgoX D3 epitope is depicted in yellow. **e** Competition analysis of CgoX mAb. Binding of DyLight-649-conjugated anti-CgoX mAb D3 to rCgoX was competed for with different concentrations of unconjugated, indicated mAbs and analysed by ELISA. Binding was determined by fluorescence measurement (Ex 646/Em 674). Data are presented as mean ± s.d. (*n* = 2). **f** Saturation binding curve was generated by plotting absorbance signals (OD_450nm_) of increasing amounts of anti-CgoX huMAb D3 to rCgoX coated on ELISA MaxiSorp plate using the GraphPadPrism 8.4 software. Kd was calculated by non-linear fitting and the equation for one-site binding model [Y = Bmax*X/(Kd + X)].

For epitope mapping, staggered overlapping peptide fragments spanning the entire respective antigen sequence were spotted on nitrocellulose or glass slides and analysed for binding of the corresponding antibody. A linear epitope could be defined for CgoX-D3, spanning aa 377–388 (Fig. 3a, and Supplementary Fig. 3a). Alanine scanning of the linear epitope revealed Ile_384_ and Arg_387_ as essential binding determinants for anti-CgoX-D3. The marginal inhibition by the alanine replacement of Leu_381_, Val_385_, Arg_386_ might be functionally irrelevant (Fig. 3b). Indeed, in silico analysis revealed surface exposure of Ile_384_ and Arg_387_ (Supplementary Fig. 3b) which is a prerequisite for antibody binding. A blast search revealed strong conservation of the CgoX epitope within 35,361 clinical *S. aureus* isolates, which predicts that mAb D3 should bind to CgoX in approximately 99% of clinical *S. aureus* isolates (Fig. 3c). Competitive binding experiments confirmed independent binding of protective and non- or less-protective mAbs to CgoX (Fig. 3e), suggesting distinct epitopes for protective and non-protective mAbs. The mAb CgoX-D3 showed high affinity binding to CgoX with a Kd of 60.38 pM (Fig. 3f).

**Fig. 4:**
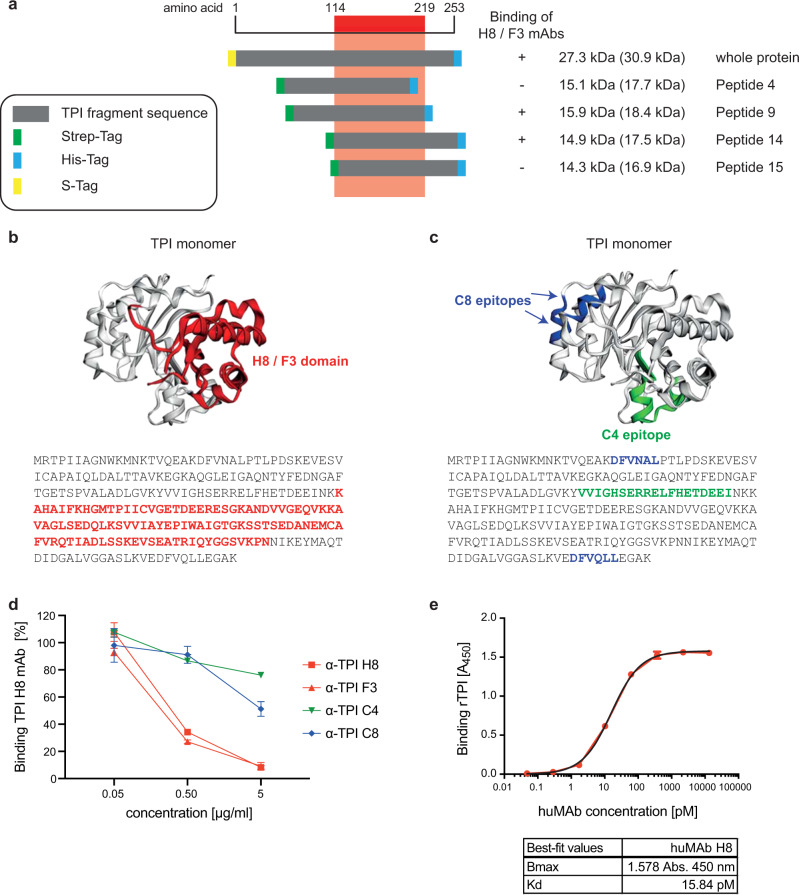
Characterisation of anti-TPI mAbs. **a** Binding domain of H8 / F3 mAbs (red) was identified by western blot analysis using recombinant His-, or Strep-tagged TPI-fragments and classified as detectable (+) or non-detectable (−) by H8/F3 mAbs in comparison to anti-Strep-tag antibodies. **b** *S. aureus* TPI structure (3m9y.pdb) was visualised by EzMol2.1 with the identified aa of the H8/F3 binding domain in red. **c** Epitopes of mAb C4 (green) and mAb C8 (blue) were identified by PepSpot technology using overlapping 15mer TPI peptides with overlapping sequences of 11 aa. **d** Competition ELISA. Binding of DyLight-649-conjugated anti-TPI mAb H8 to recombinant rTPI coated on ELISA MaxiSorp plate, was competed with unconjugated, indicated mAbs. Binding was determined by fluorescence measurement (Ex 646/Em 674). Data are presented as mean ± s.d. (*n* = 2). **e** Saturation binding curve was generated by plotting absorbance signals (OD_450nm_) of increasing amounts of anti-TPI huMAb H8 to rTPI coated on ELISA MaxiSorp plate using the GraphPadPrism 8.4 software. Kd was calculated by non-linear fitting and the equation for one-site binding model [Y = Bmax*X/(Kd + X)].

In contrast to CgoX, the protective TPI-specific mAbs H8 and F3 did not recognise a short epitope comprising less than 15 aa (Supplementary Fig. 4a). Instead, TPI fragment cloning combined with binding specificity analysis revealed that mAb H8 and -F3 bind to a polypeptide of 105 amino acid residues with K_114_ and N_219_ representing the N- and C-terminal boundary, respectively (Fig. 4a). Figure 4b shows the binding domain of TPI-H8 in a secondary structure model of TPI. The molecular surface representation of the TPI-H8 defined binding domain revealed many surface exposed aa residues potentially accessible for antibody interactions (Supplementary Fig. 4b). The amino acid residues essential for TPI-H8 binding have not yet been identified. Unlike TPI-H8, two non-protective monoclonal anti-TPI antibodies, C4 and C8 recognised distinct linear epitopes (Fig. 4c). The TPI-C4 epitope spanned aa 93–111, directly adjacent to the N-terminal boundary of the H8 mAb binding site (Fig. 4c). The mAb TPI C8 bound to two cognate epitopes at aa position 21–26 and 244–249, which were distinct from the H8 binding domain (compare Fig. 4b, c). MAb-H8 and -F3 competed with each other for binding to TPI (Fig. 4d) suggesting identical epitopes. No competition was observed between protective and non-protective anti-TPI mAbs (Fig. 4d). The mAb TPI-H8 showed high affinity binding to TPI with a Kd of 15.84 pM (Fig. 4e).

### Active immunisation with linear *S. aureus* CgoX-D3 epitope

**Fig. 5:**
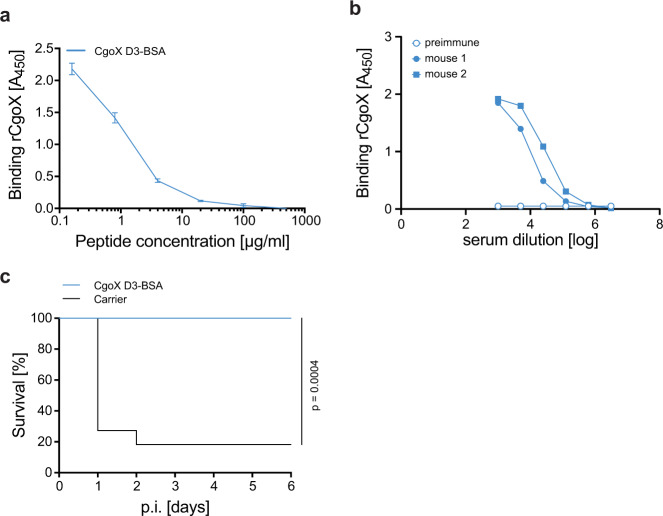
Active immunisation with anti-CgoX mAb D3 epitope peptide. **a** Competition ELISA. Binding of anti-CgoX mAb D3 to rCgoX coated on ELISA MaxiSorp plate was competed for with CgoX-D3-BSA conjugate. Binding was detected with anti-mIgG-HRP and compared to control sample (BSA). **b** Anti-CgoX-BSA IgG titer. Sera of two CgoX-D3-BSA immunised mice were collected at day 68 and analysed together with preimmune serum for anti-CgoX IgGs by ELISA. **c** Survival of mice challenged with *S. aureus* upon immunisation with CgoX-D3 epitope peptide conjugated with BSA. BALB/c mice (*n* = 11) immunised with CgoX-D3-BSA or the carrier protein BSA (black) as control group (*n* = 10), were infected i.p. with 3.3 × 10^7^ cfu *S. aureus* USA300 mixed with 5% mucin from porcine stomach. Significance was calculated by Log-rank (Mantel-Cox) test.

Functional monoclonal antibodies have become a valuable tool for immunofocusing in vaccine design, that is, in maximisation of on-target antibody responses to desired epitopes and minimisation of off-target responses^9–11^. We reasoned that the short linear peptide of the 12 aa CgoX-D3 epitope may be suitable for the induction of a more restricted and thus, functionally more precisely targeted antibody response compared to a full-length antigen. We, therefore, tested the protective mAb CgoX-D3 epitope for its potential to elicit a protective immune response when used as an active vaccine. A synthetic peptide representing the protective CgoX-D3 epitope was linked to bovine serum albumin (BSA) as protein carrier. The CgoX-D3-BSA construct specifically competed with CgoX-D3 mAb for binding to the full-length CgoX (Fig. 5a), indicating the preservation of the immunological integrity of the epitope. Mice were immunised s.c. with 80 µg of CgoX-D3-BSA in Freund´s adjuvant and boosted twice with 50 µg CgoX-D3-BSA in Freund´s incomplete adjuvant at day 23 and day 49. Indeed, CgoX-D3-BSA showed a highly significant, peptide-specific IgG response (Fig. 5b). The *S. aureus* challenge of CgoX-D3-BSA immunised mice resulted in significantly improved survival rates in the murine sepsis model (Fig. 5c). These results indicate that the CgoX-D3 epitope linked to a protein carrier suffices to elicit an effective immune response against *S. aureus*.

## Discussion

Multiple vaccine candidates for *S. aureus* infections have shown promise through preclinical development in a range of animal models. However, those that have reached late stage clinical testing have failed to demonstrate efficacy in human trials^21–23^ and occasionally aggravated the course of disease^24^. Further progress in the design of vaccines against *S. aureus* with greater precision and efficacy is needed and can be expected from increasing structural and functional characterisation of protective *S. aureus* antigens and the delineation of protective and non-protective epitopes. Herein we characterise two *S. aureus* vaccine antigens, CgoX and TPI that elicited a protective immune response against *S. aureus* infection. Two mAbs CgoX-D3 and TPI-H8 conferred protection against *S. aureus* bacteremia when used for passive immunisation. MAb CgoX-D3 specifically bound with high affinity to a short and highly conserved epitope. The 12 aa CgoX-D3 epitope conjugated to BSA as a carrier protein elicited excellent protective immunity, providing proof of principle for epitope-based *S. aureus* vaccine design. Notably, the protective mAb TPI-H8 bound to a discontinuous epitope present on an 11 kD domain within the TPI molecule, suggesting that some, but not all *S. aureus* antigens may be suitable for epitope-based vaccine design.

CgoX fulfils many criteria of a promising vaccine candidate. CgoX stands out from other vaccine candidates in that a short 12 aa epitope suffices to produce protective immunity, which is advantageous in many ways. Firstly, the peptide sequence of the D3 epitope is highly conserved in more than 35,000 clinical *S. aureus* isolates which predicts binding of mAb CgoX-D3 to almost 99% of *S. aureus* isolates (Fig. 3c). Thus, immunisation with the CgoX-D3 epitope warrants great coverage of clinically relevant *S. aureus* strains. In addition, because CgoX is essential for *S. aureus* heme biosynthesis, the emergence of deletion mutants is less likely. Secondly, when aligned with its human CgoX ortholog, the D3 epitope shows no identities (Fig. 3d), suggesting a low risk of eliciting cross-reacting antibodies. Indeed, mAb CgoX-D3 did not recognise human CgoX (Fig. 2e). Thirdly, the linear CgoX D3 epitope linked to BSA elicited a statistically greater protective immune response (*p* = 0.0004) when compared to the full-length CgoX (*p* = 0.0035). Clearly, an epitope-based vaccine minimises the risk of antibody-mediated enhancement of infection when compared to a whole vaccine protein that likely elicits a broader immune response with non-protective antibodies potentially causing immunopathogenic side effects^25^. Finally, the observation that mAb CgoX-D3 provided protection when used for passive immunisation demonstrates a great protective potential of anti CgoX-D3 antibodies independent of cellular immune responses.

CgoX operates in the cytosol at the inner leaflet of the *S. aureus* membrane^15^ and is thus not accessible for mAbs. However, its expression at the cell surface suggests an additional function of CgoX, possibly at the interface with the host, which has been described for numerous cell wall anchored antigens^17^. Interestingly, the staphylococcal CgoX (EC 1.3.3.15) catalyses the oxidation of coproporphyrinogen III to coproporphyrin III, whereas in humans, mitochondrial coproporphyrinogen oxidase (CPOX) catalyses the oxidation of coproporphyrinogen to protoporphyrin-IX (EC 1.3.3.3). This divergence between Gram-positive bacteria and humans has been recently exploited for the development of selective antibacterial modalities. Specifically, the activation of staphylococcal CgoX by small molecules resulted in the accumulation of coproporphyrin III, a photoreactive molecule, which sensitised bacteria for light-based antimicrobial therapies^26^. In humans, accumulation of porphyrins due to hereditary defects of either protoporphyrin oxidase or coproporphyrinogen oxidase are known as human variegate porphyria disease and hereditary coproporphyria^27–29^. As *S. aureus* invades host cells, it will be interesting to determine whether *S. aureus* cell surface associated CgoX interferes with host cell porphyrin metabolism. Notably, commercially available human immunoglobulin preparations contain antibodies weakly recognizing the full-sized CgoX^16^. Thus, a selective bolster of the production of antibodies directed against the D3 epitope might be required to establish immunity against invasive *S. aureus* infections.

TPI as whole protein produced protective immunity against *S. aureus*. In addition, passive immunisation with TPI-H8 conferred significant protection. Thus, TPI can be considered as a *bona fide* vaccine candidate for *S. aureus*. However, we did not identify linear epitopes of TPI that were suitable for epitope-based immunisation. TPI is a crucial enzyme in the glycolytic pathway catalysing the interconversion of the triose phosphate isomers dihydroxyacetone phosphate and D-glyceraldehyde 3-phosphate. TPI-deficient *S. aureus* mutants were not obtained, suggesting that TPI is an essential housekeeping gene. In humans, TPI deficiency is a rare autosomal recessive multisystem disorder which is dominated by lifelong hemolytic anemia and severe progressive neuromuscular degeneration^30^. Like other housekeeping glycolytic proteins such as GAPDH or enolase, TPI is exposed as an anchorless protein on the bacterial cell surface and may interact with extracellular matrix proteins of the host. A single report described an interaction of TPI with plasminogen^18^. Numerous pathogenic bacterial species intervene with the plasminogen system in vitro, suggesting that pathogenic bacteria use the plasminogen system for migration across tissue barriers or for nutritional demands during infection^19^. As to *S. aureus*, fibrinogen binding proteins (FnBPs) are well known plasminogen binding proteins, where the plasminogen binding site is masked and conformationally exposed after previous binding to fibrinogen^31,32^. Although the putative extracellular function of TPI remains to be resolved, our finding that active immunisation of mice with TPI could provide protection in a *S.aureus* bacteremia model suggested that TPI acts as *S. aureus* virulence factor. *S. aureus* TPI shows significant homology to the human TPI at the protein level (Supplementary Fig. 1). In order to minimise the risk of anti-staphylococcal TPI antibodies cross-reacting with the host TPI, a short *S. aureus* specific TPI epitope would be best suited for active immunisation. Thus, whereas mAb TPI-H8 does not show cross-reactivity with human TPI and might be considered for passive immunisation, the high overall homology between *S. aureus* TPI and its human ortholog suggests only a limited advantage of an 11 kDa TPI polypeptide over the whole antigen when used for active immunisation.

The development of human vaccines against *S. aureus* infections has relied primarily on inducing high titres of opsonising antibodies mediating antibody-dependent phagocytosis and bacterial killing by neutrophils and macrophages. However, all such vaccination attempts have failed eventually in human trials^2,7,33,34^, suggesting that opsonising antibodies do not correlate with protection. Recently, the critical role of cell-mediated immunity is being appreciated for the resolution of invasive *S. aureus* infections, and also for detrimental outcomes caused by imbalanced cellular immune responses, both of which have implications for vaccine development^4,23^. These authors suggest that vaccines targeting staphylococcal toxins and virulence factors are more likely to provide a therapeutic benefit in contrast to attempts aiming at opsonising antibodies that bear the risk of skewing the cellular immune responses, for example, by induction of cytokine production. In any case, focusing of the antibody response to a small protective epitope will avoid the production of a myriad of non-protective antibodies, thereby reducing the risk of adverse immune reactions. The use of mAbs to design new vaccines have been proposed by Burton in 2002 and is now an integral part of the ‘reverse vaccinology 2.0’ concept^9–11^. Indeed, mAbs have been widely and successfully used to understand the mechanistic nature of protection induced by vaccination^35–37^.

A phase IIb/III *S*. *aureus* vaccine study investigated the effect of a vaccine targeting *S. aureus* iron-regulated surface determinant B (IsdB) on the incidence of postoperative *S*. *aureus* bacteremia and/or deep sternal wound infection in adult patients undergoing cardiothoracic surgery through postoperative day 90. The trial had to be stopped prematurely after interim analysis showed lack of efficacy as well as a higher mortality rate in the subset of vaccine recipients developing *S*. *aureus* infections^24^. Increased levels of IsdB antibodies in patients with orthopedic infections were found to correlate with increased mortality^38^, suggesting an antibody-dependent immune enhancement. Notably, immunoprotective as well as non-protective mAbs against the *S. aureus* iron-regulated surface determinant B (IsdB) were described^39^. Two noncompeting epitopes were recognised by eight protective IsdB-specific mAbs, whereas two other mAbs also specifically bound to IsdB but were not efficacious in murine infection models. Thus, immunofocusing on a protective epitope of IsdB for active immunisation might result in a better safety profile. Clearly, immunisation with full antigens elicits both protective and non-protective antibodies. In general, non-protective antibodies bear potential risks of cross-reacting with human tissue, of functionally antagonising protective antibodies by steric hindrance of binding to their cognate epitope, or of producing adverse effects including enhancement of severity of infection. Moreover, non-protective epitopes of a given antigen may be immunodominant suppressing the generation of protective antibodies against immune-recessive epitopes. Thus, the use of mAb-defined protective linear epitopes is expected to have a greater efficacy and safety profile compared to a whole antigen. Not least, several short epitopes of different *S. aureus* vaccine antigens can be easily combined on a single polypeptide for the convenient generation of multicomponent vaccines.

## Methods

### Ethics statement

Experiments were performed in accordance with the Animal Protection Law of Germany in compliance with the Ethics Committee at the University of Cologne with age- and sex-matched groups of 7–12 weeks old mice. In accordance with these rules, our study protocols received ethical approval by the North Rhine-Westphalia State Agency for Nature, Environment and Consumer Protection-Veterinary Section, Recklinghausen, Germany under the registration numbers AZ 8.87-50.10.31.08.233 and AZ 84-02.04.2014.A013.

### Mice

Female BalbC mice were purchased from Charles River, Upon arrival, mice were acclimated and fed a standard rodent diet ad libitum.

### Bacterial strains and growth conditions

*Staphylococcus aureus* wt USA300 (NR-46543, ATCC, USA) and ATCC29213 (ATCC, USA) were cultured either on Mueller Hinton (MH)- or sheep-blood agar plates (OXOID, Germany) or in Luria Bertani (LB) broth (BD Difco, Germany) at 37 °C. Plasmid cured *S. aureus* USA300 JE2 (NR-46543) and transposon mutant Δ*CgoX* in JE2 background (NR-46607, SAUSA300_RS09750) were provided by the Network on Antimicrobial Resistance in *Staphylococcus aureus* (NARSA) distributed by BEI Resources, NIAID, NIH. The mutant was cultured on selective MH agar plates or in LB broth with 10 µg/ml erythromycin (Sigma Aldrich, Germany).

*Eschericha coli* strain DH5α (Invitrogen) and BL21 (DE3) (Stratagene) were cultured on MH-agar plates or in LB broth at 37 °C. *E. coli* strains with pET29b(+) (Novagen, Merck Millipore, Germany) antigen expression constructs were cultured in presence of 50 µg/ml kanamycin (Sigma Aldrich, Germany). *E. coli* strains with pPSG-IBA5 and pPSG-IBA45 (StarGate, IBA GmbH, Germany) antigen expression constructs were cultured in presence of 100 µg/ml carbenicillin (Sigma Aldrich, Germany).

### Expression and purification of *S. aureus* antigens

CgoX (coproporphyrinogen oxidase, Q2FXA5, formerly HemY/Protoporphyrinogen oxidase N315 WP_000167542.1), TPI (triosephosphate isomerase, NP_373984.1, *tpiA* gene) and Pephyd (peptidoglycan hydrolase, AAB62278.1, *lytM* gene) were expressed in *E. coli* strain BL21(DE3) (Stratagene) as C-terminally His_6_-tagged proteins and purified. Briefly, *CgoX*, *tpiA* and *lytM* open reading frames were amplified from *S. aureus* ATCC 29213 genome by PCR using *cgoX* sense primer: 5′-CG TCC ATG GCT AAA TCA GTG GCT A-3′ and *cgoX* anti-sense primer: 5′-AACCTCGAG CAA CTC TGC GAT TAC-3′, *tpiA* sense primer: 5′-CGACC ATG GGA ACA CCA ATT ATA GC-3′ and *tpiA* anti-sense primer 5′-GCCCTCGAG TTT TGC ACC TTC TAA-3′, and *lytM* sense primer: 5′-GT TCC ATG GAG GAT GTT TTA TAC-3′ and *lytM* anti-sense primer: 5′-GTGCTCGAG TCT ACT TTG CAA GTA-3′, respectively. PCR products were cloned into pET29b(+) (Novagen, Merck Millipore, Germany) employing restriction enzymes NcoI/XhoI and transformed into *E. coli* DH5α. Positive clones were identified by colony PCR using T7 promoter primer: 5′-TAATACGACTCACTATAGGG-3′ (Biomers, Germany) and T7 terminator anti-sense primer: 5′-GTTATGCTAGTTATTGCTCAGCGG-3′, followed by sequencing. Confirmed constructs were transformed into *E. coli* strain BL21(DE3) (Stratagene) for expression as C-terminally His_6_-tagged proteins rCgoX and rTPI, carrying an additional N-terminal S-tag. *E. coli* BL21(DE3) transformed with respective expression construct were grown in LB broth at 37 °C until the culture reached an OD_600nm_ of ~0.6. Protein expression was induced with 1 mM IPTG (Isopropyl-β-D-thiogalactoside, Sigma Aldrich, Germany) for 4 h at 30 °C. Bacterial cells were harvested, washed with 1 × PBS and resuspended in 1 × PBS containing protease inhibitors (cOmplete, EDTA-free Protease Inhibitor Cocktail, Roche, Germany). Cells were disrupted mechanically using a French Press Cell K20 (Thermo Fisher Scientific, Germany) with a pressure of 20,000 PSI and bacterial debris was removed by subsequent centrifugation at 17,000 × *g*. The expression of soluble protein was assessed by SDS-PAGE and the recombinant protein was purified by affinity chromatography using prepacked HisTrap FF columns on the ÄKTA*purifier* liquid chromatography system (GE Healthcare, Germany) according to the manufacturer’s instructions. Purified proteins were dialysed against PBS with 10% glycerol and concentration was determined by Pierce BCA Protein-Assay (Thermo Fisher Scientific, Germany) according to the manufacturer’s instructions. The purity and integrity of His_6_-tagged fusion were controlled by SDS-PAGE.

The protein structure of *S. aureus* CgoX was derived from *B. subtillis* (3I6D.pdb) using Swiss-Model workspace^40^. TPI structure (3m9y.pdb) and modulated *S. aureus* CgoX protein structures were analysed by EzMol2.1^41^.

### Western blotting

SDS-PAGE was performed using the Criterion gel system (Bio-Rad, Germany) with AnykD Criterion TGX Stain-Free Protein Gels at a constant voltage of 90 V until samples entered the separation gel, followed by a constant voltage of 300 V. Gels were imaged under UV-light using the ChemiDoc Imager (Bio-Rad, Germany) mimicking Coomassie staining.

Subsequent to protein separation by SDS-PAGE, proteins were transferred to a nitrocellulose membrane (Merck Millipore, Germany) using either the Criterion Midi Tank-Blotsystem or the Transblot Turbo transfer pack (Bio-Rad, Germany) with a constant current of 2.5 A for 1 h or 7 min., respectively. Membranes were stained with Ponceau S solution (Sigma-Aldrich, Germany), blocked with blocking solution (TBST with 5% Skimmed milk powder, 2% BSA) for 1 h at room temperature and incubated with primary antibody overnight at 4 °C. HRP-conjugated anti-His_6_-tag antibody (Thermo Fisher Scientific cat. No. PA1-23024) was used in a dilution of 1:5000, mAbs and huMAbs for the detection of the respective antigen were used in a concentration of 200 ng/ml in blocking solution. Upon washing with TBST (3 × 15 min), HRP conjugated secondary goat anti-mouse IgG (Sigma-Aldrich, Germany, cat. No. A3673) or goat anti-human IgG antibody (Bio-Rad, Germany, cat. No. 172-1050) were diluted 1:3,000 and applied for 1 h at room temperature, respectively. Bound proteins were detected using chemiluminescence reagent Clarity ECL substrate (Bio-Rad, Germany). Chemiluminescence was imaged using an MF-ChemiBIS 3.2 Imager (Berthold Technologies), a ChemiDoc™ Imager (Bio-Rad, Germany) or the blot was developed with AGFA Curix (AGFA) using an X-ray film.

For Western blot analysis of eukaryotic proteins, HeLa cells (CCL2, ATCC USA) and MEFs were cultured in DMEM (Biochrom, Germany) supplemented with 10% FBS, 100 µg/ml streptomycin and 100 U/ml penicillin. THP-1 (Invivogen, France) were cultured in RPMI (Biochrom, Germany) with 10% FBS, 100 µg/ml streptomycin and 100 U/ml penicillin and incubated at 37 °C in a humidified incubator with 5% CO_2_. For protein extraction, cells were pelleted (700 × *g*, 3 min), washed twice with PBS and resuspended in RIPA buffer (50 mM Tris-HCl pH 7.5, 150 mM NaCl, 0.1% NP-40, 0.5% sodium deoxycholate, 1% SDS, 28.5 U/ml benzonase (Merck), protease inhibitor (Roche). Protein concentration was determined using Pierce BCA Protein-Assay (Thermo Fisher Scientific). Marker bands have been indicated by their individual size. Uncropped figures are available in the Source Data file. All blots derive from the same experiment and were processed in parallel.

### Active immunisation with *S. aureus* vaccine candidates

Recombinant *S. aureus* antigens were mixed with complete Freund’s adjuvant (FA) or incomplete Freund’s adjuvant (IFA) as recommended by the manufacturer (Sigma-Aldrich, Germany). Groups of 12 mice (8–9 weeks old) were immunised intraperitoneally (i.p.) with 80 µg His_6_-tag purified rCgoX and rTPI *S. aureus* proteins formulated with FA, followed by two subcutaneous (s.c.) booster immunisations at day 33 and 56 with 40 µg antigen formulated with IFA. To obtain a stable emulsion, the formulation was passed through a T-connector (Discofix-3, Braun Melsungen AG, Germany) between two syringes for at least 100 times^20^. Mice were infected intravenously (i.v.) by injection of 3 × 10^7^ cfu of *S. aureus* strain ATCC 29213 at day 64 into the tail vein. Survival was monitored for 12 days. Significance was calculated according to Log-rank (Mantel-Cox) test in comparison to control group mock immunised with BSA formulated with FA/IFA (*n* = 11).

### Analysis of antibody titers

Antigen specific antibody-titer during mouse immunisation was determined by taking blood samples in two week-intervals starting from day 14. Serum from naïve mice served as preimmune control. ELISA assays were performed using 96 well Nunc MaxiSorp plates (Thermo Fisher Scientific, Germany) coated overnight with 500 ng/well respective antigen in coating buffer (15 mM Na_2_CO_3_, 35 mM NaHCO_3_ (pH 9.6)). After washing three times with 250 µl PBST, wells were incubated with 250 µl StartingBlock T20 blocking buffer (Thermo Fisher Scientific, Germany). Subsequently, serially diluted sera or pools of sera were added to individual wells in duplicates, and plates were incubated for 2 h at room temperature. Samples were then treated with HRP-conjugated goat anti-mouse IgG (Sigma Aldrich, Germany, cat. No. A3673) at final dilution of 1:3,000 for another 2 h at room temperature and afterwards developed with TMB-substrate (BD Biosciences, Germany). Reaction was stopped by addition of 50 µl 2 N H_2_SO_4_ (Carl Roth, Germany) and absorbance was measured at 450 nm using ELISA-reader (MRX TC, Dynex Technology, Germany or Multimode Reader TrisStar LB941, Berthold Technologies).

### Generation of monoclonal antibodies

BALB/c mice (Charles River, Germany) were immunised with rCgoX or rTPI and subsequently challenged with *S. aureus* ATCC 29213 as described above. At day 10 after infection mice received 5 µg vancomycin/g bodyweight (Sigma-Aldrich, Germany). Mice were additionally boosted s.c. with 50 µg antigen without adjuvant on three consecutive days before hybridoma generation. Spleens were dissected and splenocytes were fused to Sp2/0-Ag14 myeloma cells according to the method of Milstein and Köhler^42^. Briefly, 5 × 10^7^ spleen cells were mixed with 1.5 × 10^7^ Sp2/0-Ag14 myeloma cells (DSMZ, Germany), pelleted and resuspended in 1.5 ml polyethylene glycol-solution (Sigma-Aldrich, Germany) and subsequently in 20 ml Gibco RPMI medium (Thermo Fisher Scientific, Germany). Cells were pelleted and resuspended in 5 ml FBS before supplementing with 45 ml RPMI medium, 1 × Gibco L-GlutaMAX (Thermo Fisher Scientific, Germany), 10% FBS (Biowest, USA), 10% BMCondimed H1 (Sigma-Aldrich, Germany), 1 × Gibco HAT medium supplement (Thermo Scientific, Germany) and 24 µM Gibco ß-Mercaptoethanol (Thermo Fisher Scientific, Germany). Cell suspension was maintained for 16 h in a CO_2_-incubator before centrifugation and resuspension in the HAT medium supplemented with 1.2% methyl cellulose (Sigma-Aldrich, Germany). Cell suspension was distributed into 10 cell culture dishes and incubated for 12 days in a CO_2_-incubator without touching. Visible colonies were transferred into a 96-well plate and screened for production of antigen-specific antibodies by ELISA. IgG-subclass of each monoclonal antibody was determined by using the isotyping kit IsoStrip (Roche, Germany) according to the manufacturer’s instruction.

### Antibody purification

Hybridoma cell clones producing different anti-CgoX and anti-TPI mAbs were cultured in hybridoma serum-free medium (H-SFM, Gibco, Thermo Fisher Scientific, Germany) whereas murine NS0 cells producing anti-CgoX D3 huMAb and anti-TPI H8 huMAb were cultured in DMEM (Thermo Fisher Scientific, Germany) supplemented with 5% FBS (Biowest, USA) and 200 nM methotrexate (Sigma Aldrich, Germany) at 37 °C in a humidified incubator and 5% CO_2_. Cell culture supernatant was harvested by centrifugation at 4000 × *g* at 4 °C for 10 min and sterilized by using a 0.2 µm filter. The antibodies were purified by means of protein G (mAbs) and protein A (huMAbs) (HiTrap protein G or A HP, Sigma Aldrich, Germany) affinity chromatography in an automated Profinia protein purification system (Bio-Rad, Germany). Following the manufacturer’s protocol, antibodies were eluted with 400 mM glycine at pH 2.5 and buffer exchanged to PBS using P-6 desalting cartridges (Bio-Rad, Germany). The antibody concentration was determined by measuring the UV absorbance at 280 nm. The mAb isotype was determined by using the IsoStrip Mouse Monoclonal Antibody Isotyping Kit (Roche, Germany).

### Passive immunisation

BALB/c mice (*n* = 10, 9–12 weeks old) were immunised i.p. with 100–300 µg mAb/huMAb in 300 µl PBS or only with 300 µl PBS (control). 2–3 h later mice were challenged i.p. with 1 × 10^6^ cfu *S. aureus* USA300 or 5–8 × 10^5^ cfu ATCC 29213 in 300 µl PBS-5% mucin from porcine stomach (Sigma Aldrich, Germany). The use of mucin in *S. aureus* infection model is well established to reduce *S. aureus* inoculum and thereby opening a wider therapeutic window^43^. Survival was monitored for 6 days.

### Humanisation of antibodies

Anti-CgoX mAb D3 and anti-TPI mAb H8 were humanised at ABzena (formerly known as Antitope Ltd. Cambridge, UK) according to Abzenas proprietary Composite Human Antibody technology. In brief, heavy and light chain V region genes of each mAb secreting hybridoma were sequenced, translated and analysed for amino acid residues critical for antibody conformation and binding with structurally equivalent residues from existing antibody structures and sequence databases. Potential heavy and light chain human sequences for possible inclusion in the fully humanised sequences were identified and a series of humanised heavy and light chain V regions were then designed entirely from segments of human V region sequences avoiding putative T cell epitopes. Resulting variants of humanised heavy and light chain V region genes were constructed from overlapping, synthesised oligonucleotides assembled into full length genes using the ligase chain reaction (LCR). The LCR products were amplified by PCR and suitable restriction sites were added for cloning into the Abzena’s mammalian expression vectors between an upstream cytomegalovirus immediate/early (CMVie) promoter/enhancer plus the immunoglobulin signal sequence and the immunoglobulin constant region. The heavy and light chain vectors include genomic human IgG1 and κ constant regions. The resulting humanised antibody heavy and light chain-encoding plasmids were then co-transfected into murine NS0 cells by electroporation and Protein A-purified, humanised antibodies (huMAb) were tested in antigen binding assays for specificity and subjected to EpiScreen whole antibody human T cell assays using 20 healthy volunteers as donors of blood samples. The immunogenicity of the lead humanised antibody was benchmarked against EpiScreen whole protein data for clinical-stage biologics.

### *S. aureus* proliferation upon addition of mAb

An overnight culture of respective *S. aureus* strain JE2 was diluted 1:100 and grown in LB medium at 37 °C until an OD_600nm_ of 0.3–0.6. Cells were harvested (4000 × *g*, 10 min) and washed with PBS. 3 × 10^8^ bacteria/ml were incubated with the indicated antibody concentration for 20 min at 37 °C in PBS. Bacteria-antibody suspensions were diluted 1:1000 in 200 µl LB medium and measured every 5 min in a Tecan Infinite M Plex at OD_600nm_ for 500 min at 37 °C.

### Epitope identification

The epitope of the anti-TPI mAb C8 was identified by Pepspot technology (JPT GmbH, Berlin, Germany) using synthesised TPI peptide-fragments of 15 aa with overlapping sequences of 11 aa according to the manufacturer’s instruction. The epitope of anti-CgoX mAb D3 was identified by microarray analyses performed by the Pepperprint GmbH (Germany) using synthesised peptide-fragments of 13 amino acids with overlapping sequences of 12 aa.

The discontinuous epitope of mAbs anti-TPI H8 and F3 was identified by western blot analysis using TPI-peptide fragments. TPI open reading frame was amplified from *S. aureus* ATCC 29213 genome by PCR using different sets of primers. For peptide 4 (aa 57–195): Sense primer: 5′-AGCGGCTCTTCAATGAAAGCACAAGGTTTAGA AATCG-3′ and anti-sense primer: 5′-AGCGGCTCTTCTCCCAGTTTGACGTACAAAT GCACAC-3′, for peptide 9 (aa 73–219): Sense primer: 5′-AGCGGCTC TTCAATGAATGGTGCGTTCACAGGTGAAAC-3′ and anti-sense primer: 5′-AGCGGCTCTTCTCCCGTTAGGTTTAACACTACCACC-3′, for peptide 14 (aa 114–253): Sense primer: 5′-AGCGGCTCTTCAATGAAAGCGCACGCTATTTTC-3′ and anti-sense primer: 5′-AGCGGCTCTTCTCCCTTTTGCACCTTCTAACAATTGTACG-3′ and for peptide 15 (aa 120–253): Sense primer: 5′-AGCGGC TCTTCAATGAAACATGGAATGACTCCA-3′ and anti-sense primer: 5′-AGCGG CTCTTCTCCCTTTTGCACCTTCTAACAATTGTACG-3′. PCR products were cloned into pPSG-IBA5 and pPSG-IBA43 vector employing the StarGate combinatorial cloning system via ENTRY cloning for fusion to a N-terminal Strep-tag II (pPSG-IBA5) and a N- terminal Strep-tag II and a C-terminal His_6_-tag (pPSG-IBA45; IBA GmbH, Germany). Constructs were transformed into *E. coli* DH5α and validated by sequencing using ENTRY sense prime 5′-GCGAAACGATCCTCGAAGC-3′ and antisense prime 5′- CCCCTGATTCTGTGGATAACCG-3′. Confirmed constructs were cloned into pPSG-IBA5 or pPSG-IBA45 and transformed into *E. coli* strain BL21(DE3) (Stratagene, Germany) for expression. For this, bacteria expressing the respective peptide were grown in LB medium containing carbenicillin (100 µg/ml) for pPSG-IBA43 constructs at 37 °C until the culture reached an OD_600nm_ of ~0.6. The protein expression was induced with 1 mM IPTG for 4 h at 30 °C. Bacteria cells were harvested and lysed with BugBuster Protein Extraction Reagent (Merck Millipore, Germany) according to the manufacturer’s instruction. After the lysis of *E. coli* cells expressing TPI fragments 4, 9, 14, and 15, respectively, preparations of inclusion bodies (IB) as well as cytoplasmic fractions (CF) were blotted on nitrocellulose membrane and binding of anti-TPI mAb H8 to immobilised peptides was shown using HRP-conjugated goat anti-mouse IgG secondary antibody (Sigma-Aldrich, Germany, cat No. A3673) at final dilution of 1:3000. The expression of the peptides was verified by detection with anti-Strep-tag-HRP (IBA GmbH, Germany, cat. No. 2-1509-001) at final dilution of 1:4000.

### Fine specificity analysis of mAb CgoX D3 binding by alanine scan

Biotinylated 12mer epitope peptides of anti-CgoX mAb D3, were synthesised by JPT GmbH, Germany, in which consecutive amino acids were individually replaced by alanine. Pierce Streptavidin coated plates (Thermo Fisher Scientific, Germany) were incubated first with the biotinylated peptides for immobilisation and washed according to the manufacturer’s instructions. Plates were then incubated with mAb CgoX D3 and with goat anti-mouse IgG-HRP (Sigma-Aldrich, Germany, cat. No. A3673) at final dilution of 1:3000. For detection TMB-substrate (BD Biosciences, Germany) was added and the reaction was stopped by 2 N H_2_SO_4_ (Carl Roth, Germany). The absorbance was measured at 450 nm using ELISA-reader Multimode Reader TrisStar LB941 (Berthold Technologies, Germany).

### Conservation of CgoX D3 cognate epitope in *S. aureus* clinical isolates

To determine the epitope frequencies, we data mined the RidomSeqsphere core genome multi locus sequence typing (cgMLST) Nomenclature Server (cgMLST.org) for counts for all alleles of locus SACOL1887 (CgoX). Allele sequences were then translated into amino acids and aligned using Clustal Omega (https://www.ebi.ac.uk/Tools/msa/clustalo/).

### Competition analysis of mAb binding to epitopes

To determine if mAbs recognise overlapping epitopes on their corresponding antigen, competition-ELISA were performed. 96-well Nunc MaxiSorp plates (Thermo Scientific, Germany) were coated with 500 ng/well of rCgoX or rTPI, respectively, in PBS at 4 °C over night. Afterwards, the plate was blocked with StartingBlock T20 (PBS) Blocking Buffer (Thermo Scientific, Germany) for 60 min at RT. Binding of DyLight-649 conjugated mAbs to recombinant corresponding antigen was analysed in the presence of increasing concentrations of competing unconjugated mAbs. Binding was determined by fluorescence measurement (Ex 646/Em 674) with microplate reader Infinite M1000 (Tecan, Switzerland).

### Epitope-specific competition

To evaluate the competitive capacity of the CgoX-D3 epitope, 500 ng/well of the full-length rCgoX was coated on ELISA MaxiSorp plate in PBS at 4 °C over night. The plate was blocked with StartingBlock T20 (PBS) blocking buffer (Thermo Scientific, Germany) for 1 h at RT. The competitor epitope conjugate (CgoX-D3-BSA) was pre-diluted in a 96-well Nunc MaxiSorp plate (Thermo Scientific, Germany) from 500 µg/ml to 0.8 µg/ml and the dilutions were added to the antigen-coated ELISA plate in a total volume of 50 µl per well. Thereafter, 50 µl of anti-CgoX mAb D3 (1 ng/well) was added and incubated for 2 h. The secondary antibody (goat anti-mouse or goat anti-human-HRP) was diluted 1:3000. All dilutions were prepared in Starting Block™ T20 (PBS) blocking buffer. Between the incubation steps the plate was washed 3× with 1× PBST. For the enzymatic reaction TMB substrate reagent set (BD Biosciences) was used according to the manufacturer’s instructions. The absorbance was measured at 450 nm using ELISA-reader Multimode Reader TrisStar LB941 (Berthold Technologies, Germany).

### Active immunisation with CgoX D3 epitope-BSA conjugate

BALB/c mice (*n* = 2) were immunised with 80 µg peptide-conjugate CgoX D3-BSA emulsified with FA on day 0 and boosted twice with 50 µg of the peptide-conjugate emulsified with IFA on day 23 and 49 according to recommmendations of Stills and therefore avoiding i.p. immunisation^44^. Blood was collected prior to and post immunisation at day 66. Sera were analysed by ELISA for antigen-specific IgG titer.

Groups of 11 BALB/c mice (8–9 weeks old) were initially s.c. immunised with 50 µg CgoX-D3-BSA conjugate formulated with FA, followed by two s.c. booster immunisations at day 24 and 47 with 50 µg peptide-BSA conjugate formulated with IFA, before i.p. infection on day 61 with 3.5 × 10^7^ cfu *S. aureus* USA300 mixed with PBS-5% mucin from porcine stomach (Sigma, Germany). Survival was monitored for 6 days. Significance was calculated according to Log-rank (Mantel-Cox) test in comparison to control group immunised accordingly with BSA formulated with FA/IFA (*n* = 10).

### Statistical analyses

All experiments were conducted a minimum of two times, representative data are shown in graphs. Data collected was plotted and analysed using GraphPad Prism software with two-sided tests. Statistical significance of survival data were calculated using log-rank (Mantel-Cox) test, exact *p*-values are given in graphs.

### Reporting summary

Further information on research design is available in the Nature Research Reporting Summary linked to this article.

## Supplementary information

Supplementary Information

Reporting Summary

## Data Availability

All data generated during this study are available from the corresponding author upon reasonable request.
