# Supplementary material for: Epitope-specific immunity against Staphylococcus aureus coproporphyrinogen III oxidase
Source: NPJ Vaccines. 2021 Jan 18;6:11. doi: 10.1038/s41541-020-00268-2 (PMC7813823; doi:10.1038/s41541-020-00268-2)
Supplement: Supplementary file 1 — Supplementary Information [file 41541_2020_268_MOESM1_ESM.pdf]

Supplementary Figures

Coproporphyrinogen oxidase (CgoX) protein sequence

```
Staphylococcus aureus 1    MTKSVAIIGAGITGLSSAYFLKQQDPNIDVTIFEASNRPGGKIQSYRK-DGYMIELGPESYLGRKTI-MTELAKDIGLEQ  78
Homo sapiens PPOX     1    MGRTVVVLGGGISGLAASYHLSRAPCPPKVVLVESSERLGGWIRSVRGPNGAIFELGPRGIRPAGALGARTLLLVSELGL  80
Mus musculus PPOX     1    MGRTVIVLGGGISGLAASYHLIRGPSPPKVILVEGSKRLGGWIRSIRGSDGAIFELGPRGIRPAGALGARTLLLVSELGL  80

Staphylococcus aureus 79   DIVTNTTGQSYIFAKNKLYPIPGGSIMGIPTDIKPFVTTKLISPLGKLRAGLDLIKKPIQMQDGDISVGAFFRARLGNEV  158
Homo sapiens PPOX     81   DSEVLPVRGDHPAAQNR----FLYVGGALHALPTGLRGLLRPSPPFS--------------KPLFWAGLRELTKPRGKEP  142
Mus musculus PPOX     81   ESEVLPVRGDHPAAQNR----FLYVGGTLHPLPSGLRGLLRPSPPFS--------------KPLFWAGLRELLKPRGKEP  142

Staphylococcus aureus 159  LE-------NLIEPLMGGIYGTDIDKLSLMSTFPNFKEKEEAFGSLIKGMKDEKNKRL------KQRQLYPGAPKGQFK-  224
Homo sapiens PPOX     143  DETVHSFAQRRLGPEVASLAMDSLCRGVFAGNSRELSIRS-CFPSLFQAEQTHRSILLGLLLGAGRTPQPDSALIRQALA  221
Mus musculus PPOX     143  DETVHSFAQRRLGPEVASLAMDSLCRGVFAGNSRELSIRS-CFPSLFQAEQTHRSILLGLLLGAGQSPQPDSSLIRQARA  221

Staphylococcus aureus 225  ------QFKHGLSSFIEALEQDVKNKGVTIRYNTSVDDIITSQKQYKIVYSNQQEDVFDGVLVTTPHQVFLNWFGQD--P  296
Homo sapiens PPOX     222  ERWSQWSLRGGLEMLPQALETHLTSRGVSVLRGQPVCGLSLQAEGRWKVSLRDSSLEADHVISAIPASVLSELLPAEAAP  301
Mus musculus PPOX     222  ERWSQWSLRGGLEVLPQALHNHLASKGVTVLSGQPVCGLSLQPEGRWKVSLGDSSLEADHIISAIPASELSKLLPAEAAP  301

Staphylococcus aureus 297  AFDYFKTMDSTTVATVVLAFDEKDIENTYDGTGFVIARTSDTDITACTWTSKKWPFTTPEGKVLIRAYVGKPGD-----T  371
Homo sapiens PPOX     302  LARALSAITAVSVAVVNLQYQGAHLPV--QGFGHLVPSSEDPGVLGIVYDSVAFPEQDGSPPGLRVTVMLGGSWLQTLEA  379
Mus musculus PPOX     302  LARILSTIKAVSVAVVNLQYRGACLPV--QGFGHLVPSSEDPTVLGIVYDSVAFPEQDGNPPSLRVTVMLGGYWLQKLKA  379

Staphylococcus aureus 372  VVDDHTDNELVSIVRRDLSQMMTFKGDPEFTIVNRLPKS-MPQYHVGHIQQIRQIQAHIKQTYPRLRVTGASFEAVGLPD  450
Homo sapiens PPOX     380  SGCVLSQELFQQRAQEAAATQLGLK-EMPSHCLVHLHKNCIPQYTLGHWQKLESARQFLTAHRLPLTLAGASYEGVAVND  458
Mus musculus PPOX     380  AGHQLSPELFQQQAQEAAATQLGLK-EPPSHCLVHLHKNCIPQYTIGHWQKLDSAMQFLTAQRLPLTLAGASYEGVAVND  458

Staphylococcus aureus 451  CITQGKVAAEEVIAEL---  466
Homo sapiens PPOX     459  CIESGRQAAVSVLGTEPNS  477
Mus musculus PPOX     459  CIESGRQAAVAVLGTESNS  477
```

Triose phosphate isomerase (TPI) protein sequence

```
Staphylococcus aureus 1    M---------------------------------------RTPIIAGNWKMNKTVQEAKDFVNALPTLPDSKEVESVIC  40
Homo sapiens          1    MAEDGEEAEFHFAALYISGQWPRLRADTDLQRLGSSAMAPSRKFFVGGNWKMNGRKQSLGELIGTLNAAKVPADTEVVCA  80
Mus musculus          1    ------------------------------------MAPTRKFFVGGNWKMNGRKKCLGELICTLNAANVPAGTEVVCA  43

Staphylococcus aureus 41   APAIQLDALTTAVKEGKAQGLEIGAQNTYFEDNGAFTGETSPVALADLGVKYVVIGHSERRELFHETDEEINKKAHAIFK  120
Homo sapiens          81   PPTAYIDFARQKLDPK----IAVAAQNCYKVTNGAFTGEISPGMIKDCGATWVVLGHSERRHVFGESDELIGQKVAHALA  156
Mus musculus          44   PPTAYIDFARQKLDPK----IAVAAQNCYKVTNGAFTGEISPGMIKDLGATWVVLGHSERRHVFGESDELIGQKVSHALA  119

Staphylococcus aureus 121  HGMTPIICVGETDEERESGKANDVVGEQVKKAVAGLSEDQLKSVVIAYEPIWAIGTGKSSTSEDANEMCAFVRQTIADLS  200
Homo sapiens          157  EGLGVIACIGEKLDEREAGITEKVVFEQTKV-IADNVKDWSK-VVLAYEPVWAIGTGKTATPQQAQEVHEKLRGWLKSNV  234
Mus musculus          120  EGLGVIACIGEKLDEREAGITEKVVFEQTKV-IADNVKDWSK-VVLAYEPVWAIGTGKTATPQQAQEVHEKLRGWLKSNV  197

Staphylococcus aureus 201  SKEVSEATRIQYGGSVKPNNIKEYMAQTDIDGALVGGASLKVEDFVQLLEGAK  253
Homo sapiens          235  SDAVAQSTRIIYGGSVTGATCKELASQPDVDGFLVGGASLKPE-FVDIINAKQ  286
Mus musculus          198  NDGVAQSTRIIYGGSVTGATCKELASQPDVDGFLVGGASLKPE-FVDIINAKQ  249
```

A = highly conserved amino acids

A = less conserved amino acids

A = gaps in alignment

Supplementary Figure 1: Homology alignment of staphylococcal CgoX, TPI. Amino acid (aa) sequence-alignment of CgoX- and TPI- proteins of *S. aureus*, *H. sapiens* and *M. musculus* using Cobalt RID (NCBI). Identical aa are depicted in red and less conserved aa in black. Gaps in sequence alignment are marked in blue.

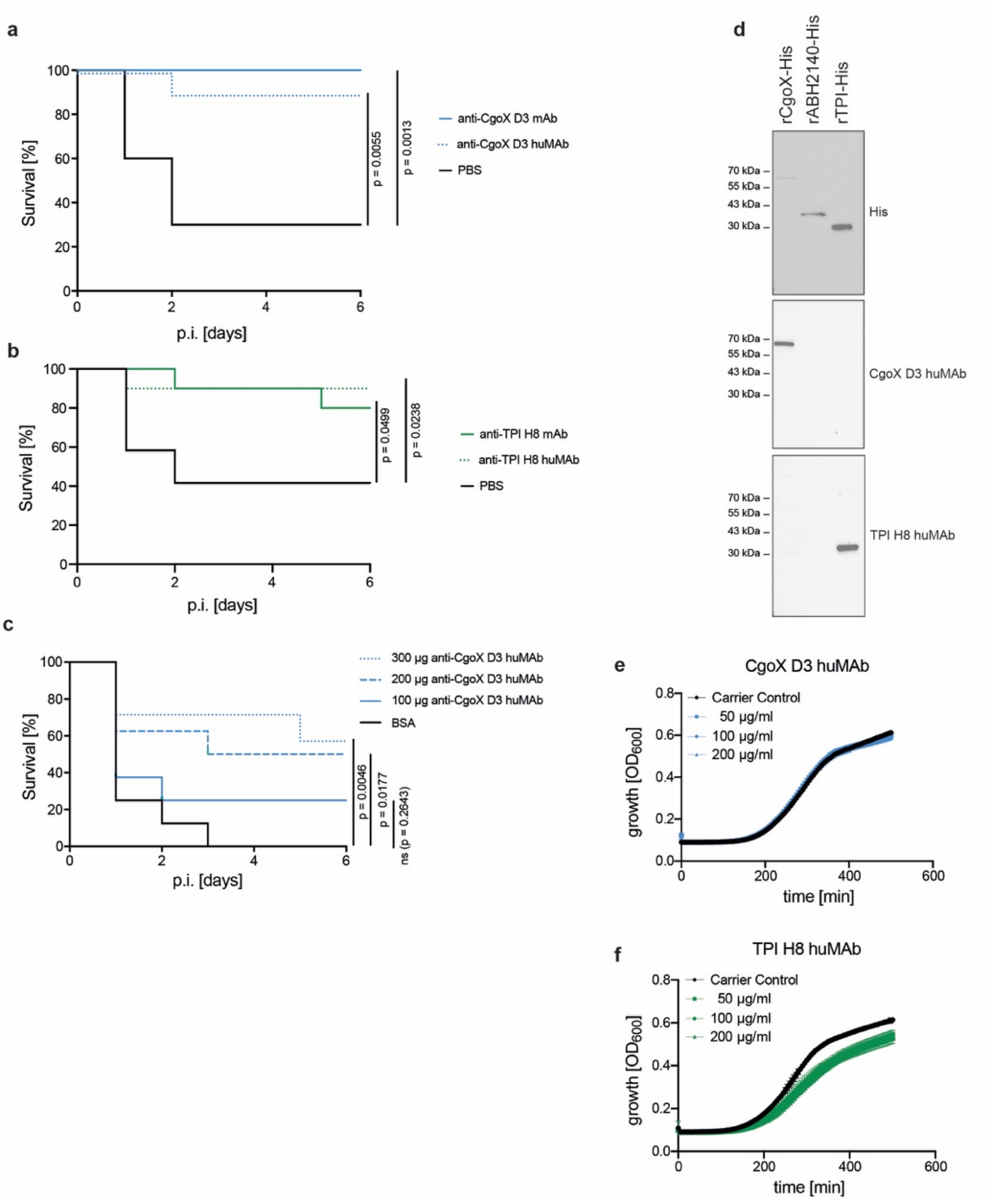

Supplementary Figure 2: Passive immunisation with huMAbs against *S. aureus* infection. BALB/C mice (n = 10) were immunised with 200 µg mAbs or huMAbs directed against CgoX (a, anti-CgoX D3) or TPI (b, anti-TPI H8) in 200 µl PBS or received PBS alone (control). c) For dose response analysis BALB/c mice (n = 10) were immunised with 100 µg, 200 µg or 300 µg huMAb anti-CgoX D3 or 200 µg bovine serum albumine (BSA) in 200 µl PBS i.p. Mice were subsequently challenged i.p. with 1 x 10^6 cfu USA300. Significance was calculated according to Log-rank (Mantel-Cox) test in comparison to control group immunised with PBS. d) Specific binding of huMAbs to their corresponding recombinant antigen. 2 µg of each His_6-tagged, purified antigen was blotted on nitrocellulose and analysed by Western blot. e, f) Growth curves (OD_{600}) of *S. aureus* strain USA300 pre-incubated with the indicated huMAbs (n = 4 biological replicates).

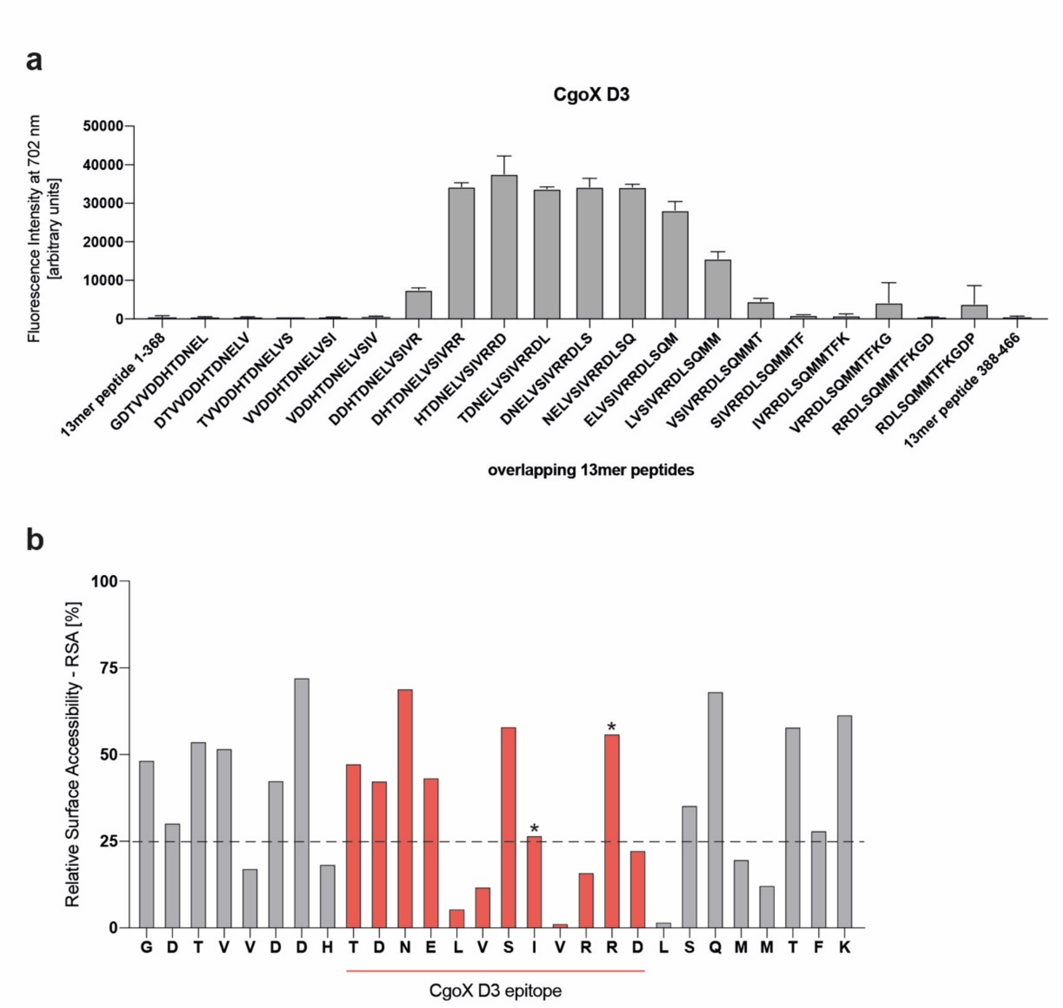

Supplementary Figure 3: Epitope analysis of anti-CgoX mAb D3. a) Linear 13mer antigen peptides with overlapping sequences of 12 amino acids were spotted on a glass slide in duplicate and binding of anti-CgoX mAb D3 was analysed by incubation with anti-mIgG-DyLight680 conjugate and subsequent fluorescence measurement (Ex 682 ⁄ Em 702). b) Surface accessibility analysis of CgoX-D3 epitope. AA of *S. aureus* CgoX was analysed for surface exposure using program NetsurfP displaying relative solvent accessibility (RSA) with > 25 % as universally accepted threshold for exposed aa[1]. Epitope region is coloured in red.

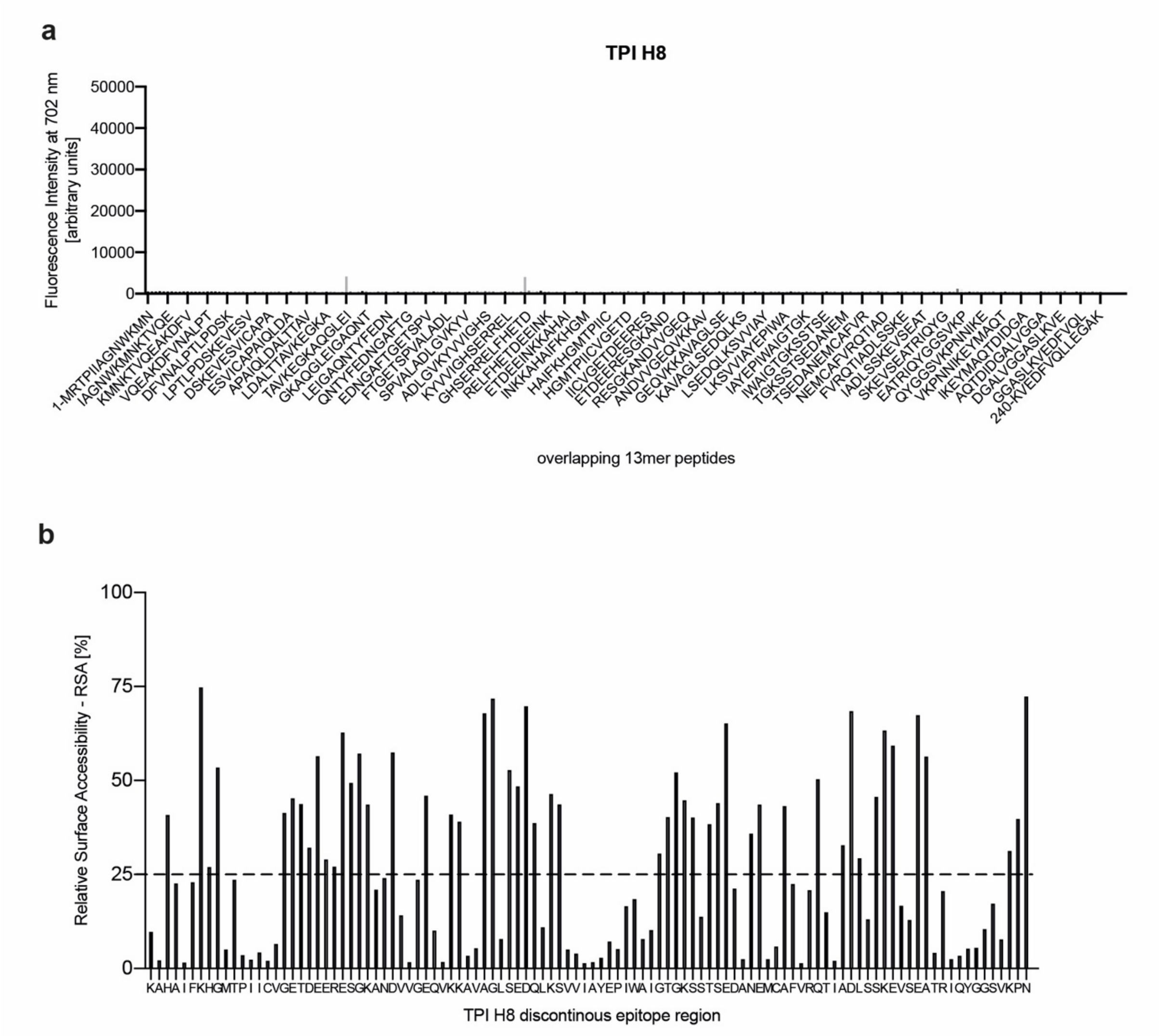

Supplementary Figure 4: Analysis of anti-TPI mAb H8 binding region. a) Linear 13mer antigen peptides with overlapping sequences of 12 amino acids were spotted on a glass slide in duplicate and binding of anti-TPI mAb H8 was analysed by incubation with anti-mIgG-DyLight680 conjugate and subsequent fluorescence measurement (Ex 682 ⁄ Em 702). b) Surface accessibility analysis of TPI-H8 binding region. AA of *S. aureus* TPI was analysed for surface exposure using program NetsurfP displaying relative solvent accessibility (RSA) with >25 % as universally accepted threshold for exposed aa[1].

Figure 1a

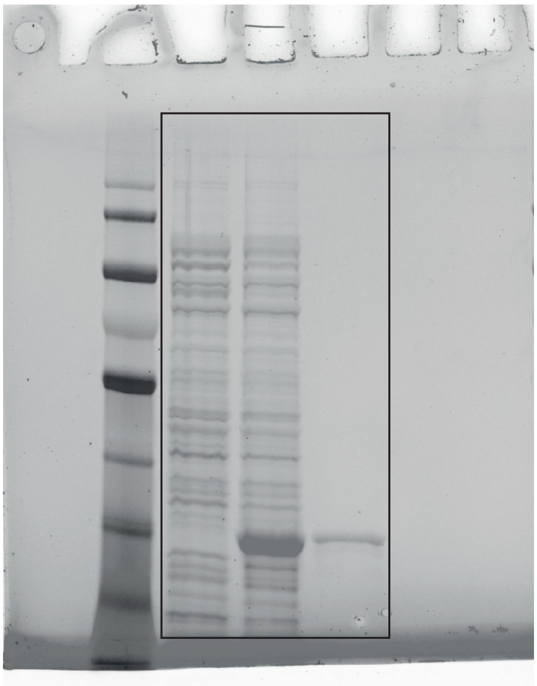

Figure 1d

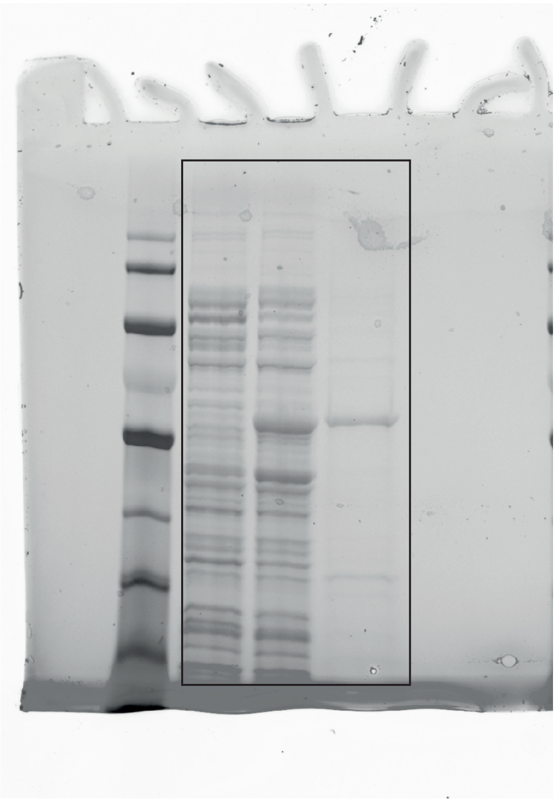

Figure 1b

Figure 1e

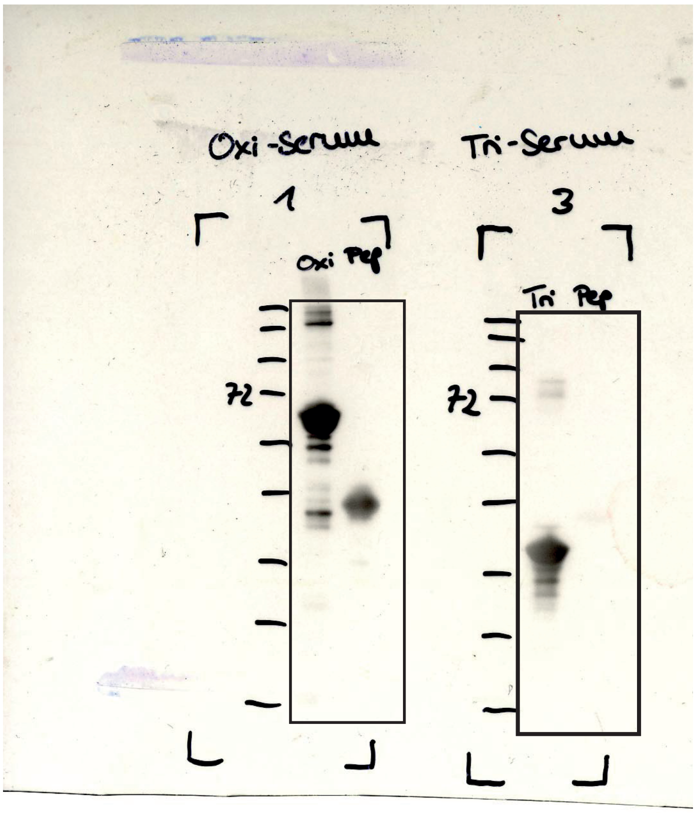

Figure 2d

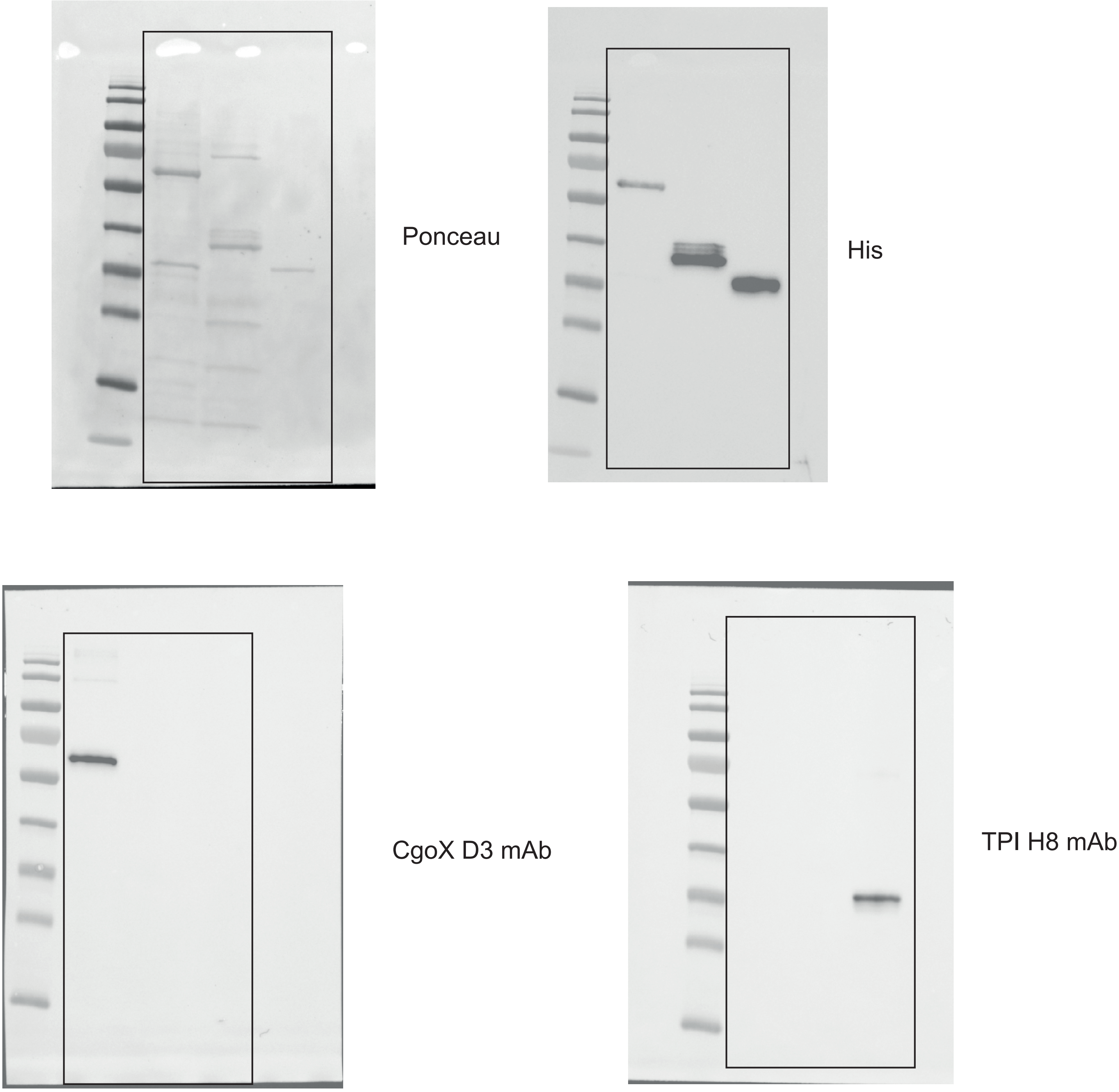

Figure 2e

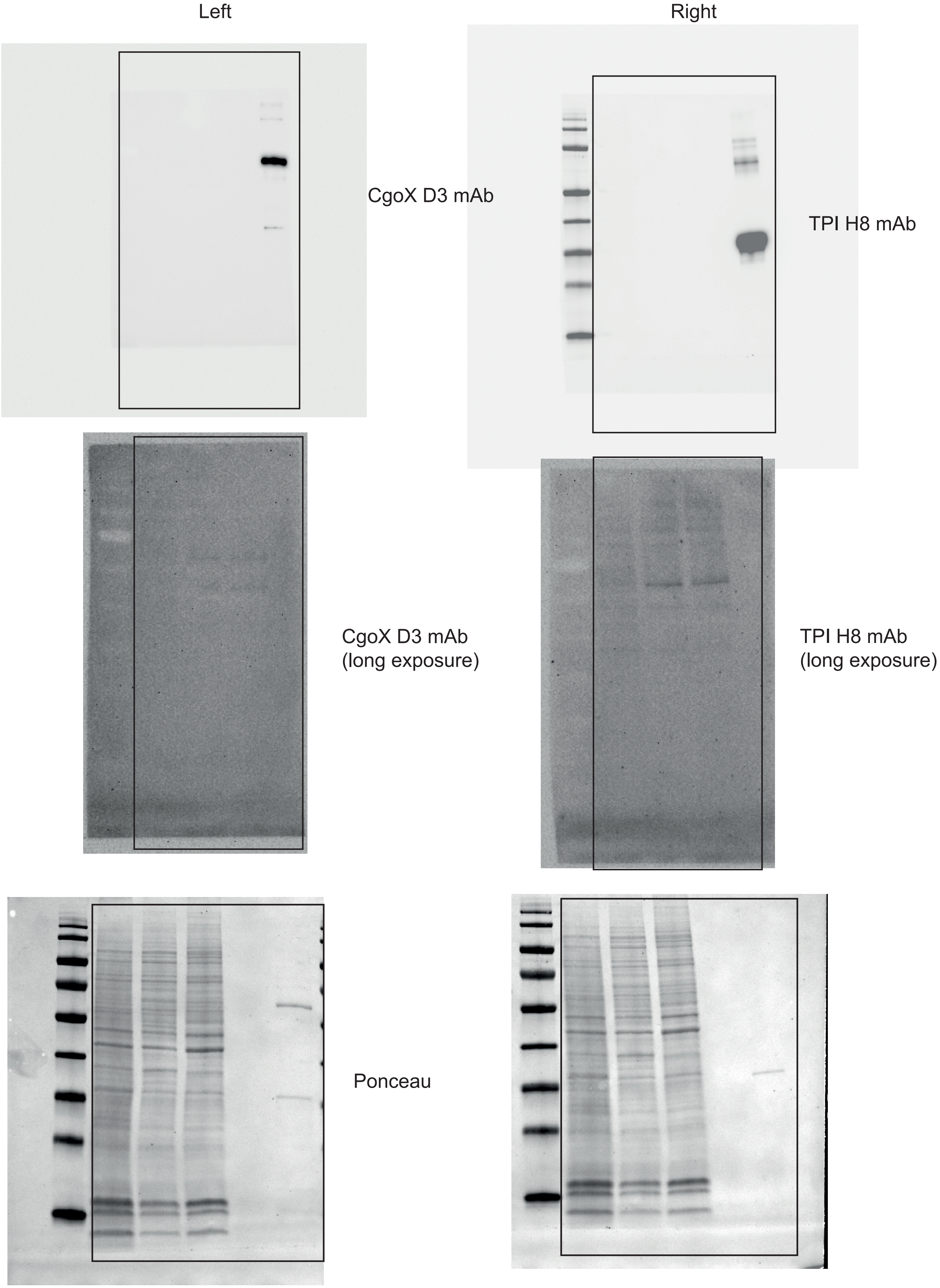
Left
Right
CgoX D3 mAb
TPI H8 mAb
CgoX D3 mAb
(long exposure)
TPI H8 mAb
(long exposure)
Ponceau

Supplementary Figure 2d

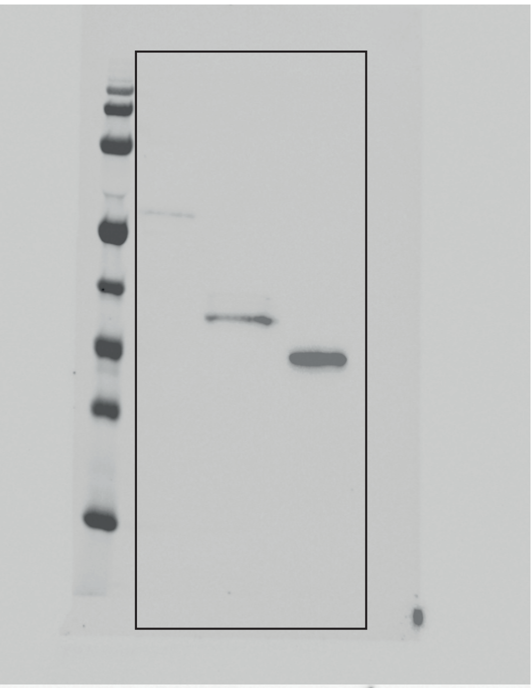

His

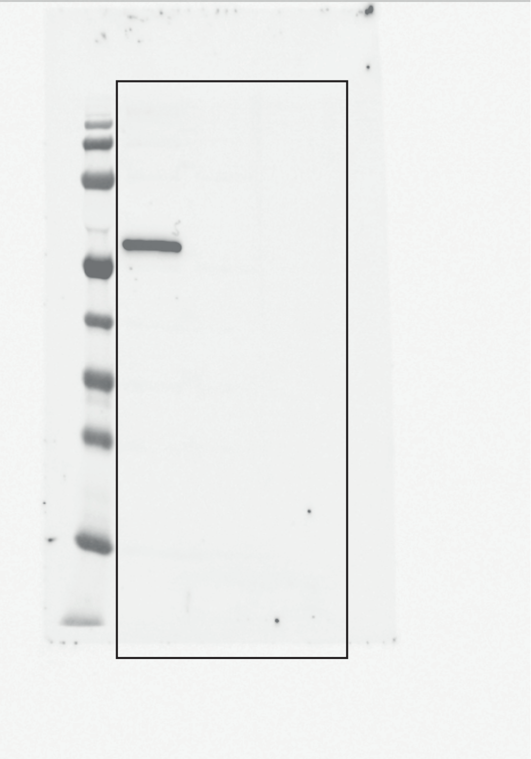

CgoX D3 huMAb

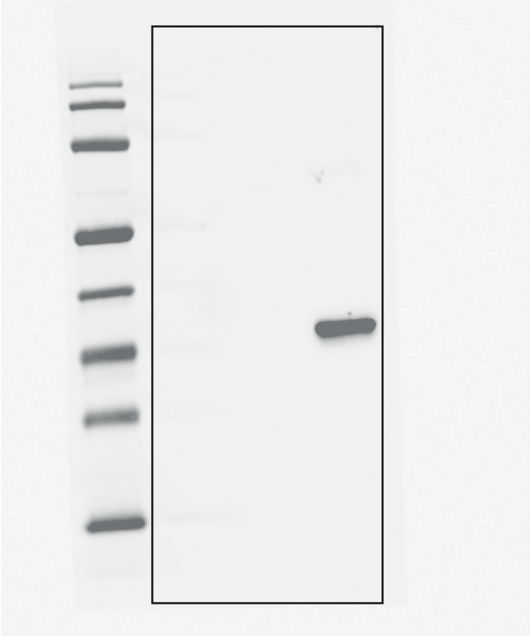

TPI H8 huMAb

Supplementary References

1. Wu, W., Wang, Z., Cong, P. & Li, T. Accurate prediction of protein relative solvent accessibility using a balanced model. ***BioData Min.*** **10**, 1 (2017).
